# Epigenetic Markers of Cell Division and Ageing in Relation to Breast Cancer Survival

**DOI:** 10.1002/ijc.70618

**Published:** 2026-06-27

**Authors:** Luca Li, Elaheh Zarean, Danmeng Lily Li, Yiyue Zhu, Shuai Li, Enes Makalic, Catriona McLean, Graham G. Giles, Roger L. Milne, Melissa C. Southey, Pierre‐Antoine Dugué

**Affiliations:** ^1^ Precision Medicine, School of Clinical Sciences at Monash Health, Monash University Clayton Victoria Australia; ^2^ Centre for Epidemiology and Biostatistics, Melbourne School of Population and Global Health, The University of Melbourne Parkville Victoria Australia; ^3^ Department of Data Science and AI, Faculty of Information Technology Monash University Clayton Victoria Australia; ^4^ Anatomical Pathology Alfred Health, The Alfred Hospital Melbourne Victoria Australia; ^5^ Cancer Epidemiology Division Cancer Council Victoria Melbourne Victoria Australia

**Keywords:** ageing, breast cancer, cancer survival, DNA methylation, epigenetics

## Abstract

Breast cancer remains a major challenge to public health. Biomarkers may be useful to improve prediction of breast cancer survival. Several epigenetic markers of cell division and ageing based on DNA methylation have been proposed. In this study, we measured these epigenetic markers in breast tumours and assessed their prognostic value. We used genome‐wide DNA methylation data measured in 1992 breast cancer tumours from the Melbourne Collaborative Cohort Study and publicly available datasets. We calculated four markers of cell division (*epiTOC2*, *stemTOC*, *MiAge* and *CellDRIFT*), two markers of chronological age (*Horvath age* and *BTEC*), and four markers of biological age (*PhenoAge*, *GrimAge*, *MRscore* and *DunedinPACE*). Cox regression models were used to assess the associations of age‐adjusted epigenetic markers with 5‐year overall survival, with adjustment for clinical variables. Effect modification by estrogen receptor (ER) status and molecular subtype was also investigated. After adjustment for age and stratification by study, higher levels of cell division markers were associated with poorer survival (e.g., *epiTOC2*: per one‐standard‐deviation increase, hazard ratio [HR] = 1.14, 95% CI: 1.03–1.27), whereas higher chronological age markers were linked to better prognosis (e.g., *Horvath age*: HR = 0.70, 95% CI: 0.61–0.82). Epigenetic markers of biological age showed variable associations. These associations were partly explained by the main clinicopathological variables at diagnosis and varied across subtypes. Our study revealed associations of several epigenetic markers with breast cancer survival. The associations were quite weak, suggesting these markers may have limited prognostic value. The varying associations observed among subtypes may reflect underlying biological differences that should be further investigated.

## What's New?

Epigenetic changes influence tumour behaviour, but whether DNA methylation‐based markers of cell division and ageing meaningfully predict breast cancer survival remains uncertain. Here, researchers analyzed genome‐wide DNA methylation data from nearly 2000 breast tumours to evaluate 10 epigenetic markers and their associations with 5‐year survival. Higher levels of cell‐division markers were linked to poorer survival, whereas chronological ageing markers were associated with better outcomes. Associations varied across breast cancer subtypes. However, effects were generally modest and partly explained by established clinical factors. The findings suggest these markers reflect important tumour biology but offer limited additional prognostic value beyond standard measures.

## Graphical abstract

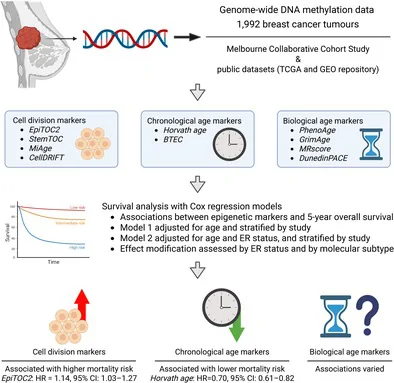

**Abbreviations**
CI: confidence interval
CpG: 5′—cytosine—phosphate—guanine—3’
DNA: deoxyribonucleic acid
ENmix: empirical normal mixture background correction
ER: estrogen receptor
GEO: gene expression omnibus
HER2: human epidermal growth factor receptor 2
HR: hazard ratio
IDAT: illumina data file
IQR: interquartile range
MCCS: melbourne collaborative cohort study
OS: overall survival
PR: progesterone receptor
SD: standard deviation
TCGA: the cancer genome atlas
TNBC: triple‐negative breast cancer

## 1 Introduction

Breast cancer is the most commonly diagnosed cancer among females and one of the five leading causes of cancer‐related death in the world [1]. It is commonly classified molecularly based on the presence or absence of well‐defined immunohistochemical markers. These markers mainly include estrogen receptors (ERs), progesterone receptors (PRs), and human epidermal growth factor receptor 2 (HER2), and others may be used such as Ki‐67, a proliferating cell nuclear antigen [2]. These markers define molecular subtypes that are associated with different prognoses, with Luminal A tumours generally having the most favourable outcomes and triple‐negative tumours the least favourable [2, 3].

Beyond molecular subtypes, tumour behaviour can also be influenced by epigenetic changes, which regulate gene expression without altering the deoxyribonucleic acid (DNA) sequence [4]. According to the epigenetic progenitor model of cancer, tumourigenesis begins with an epigenetic alteration within a given tissue, followed by an initiating mutation in gatekeeper genes within the epigenetically disrupted progenitor cells, and increased epigenetic and genetic plasticity in descendant cells [5]. One global epigenetic alteration observed across all tumours is aberrant DNA methylation [5]. Changes to the status of DNA methylation at CpG sites can occur due to ageing, cell division, environmental factors, physiological stimuli, genetic mutations or random stochastic errors in the maintenance of DNA methylation [6]. Because these changes reflect diverse biological processes, DNA methylation has been used to develop markers of cell division [7, 8, 9, 10], chronological age [11, 12], biological age [13, 14, 15, 16] and tumour purity [17].

Several DNA methylation‐based markers of cell division have been developed to trace cell division history in tissues. The *epiTOC2* model directly estimates the accumulated number of stem cell divisions in a tissue. It was developed to predict cancer risk, having the ability to differentiate precancerous lesions from normal tissues [5, 7]. A more recently developed cell division marker, *stemTOC*, improves on *epiTOC2* by reducing confounding from chronological age [8]. Like *epiTOC2* and *stemTOC*, *MiAge* estimates mitotic history from replication‐related errors in the inheritance of DNA methylation patterns [7, 8, 9]. *MiAge* has been found to be associated with survival from several cancer types when measured in tumour tissues [9]. *CellDRIFT* is a novel signature of cell replication. It increases with chronological age across multiple tissues, distinguishes between normal and cancer tissue, and was reported to be associated with breast cancer survival in a recent study, albeit based on a small sample [10].

Chronological age markers were designed to predict chronological age in tissues. *Horvath age* is a multi‐tissue predictor of chronological age [11], and several studies have investigated *Horvath age* measured in tumours as a cancer prognosis marker [18, 19, 20, 21]. *BTEC*, developed specifically for breast tissue, aimed to predict chronological age more accurately in breast tissue and to detect abnormal molecular changes induced by tumour development [12]. Biological ageing markers provide information beyond chronological age. *PhenoAge* was found to be associated with numerous health outcomes including all‐cause mortality, physical functioning and risk of cancer, cardiovascular disease and Alzheimer's disease [13]. *GrimAge* is generally considered to be one of the best ageing markers, showing strong associations with numerous diseases [14]. *MRscore* was developed to predict all‐cause mortality with a small number of CpGs [15]. *DunedinPACE* estimates the pace of ageing and has shown associations with mortality and other health‐related traits [16]. Although these biological age markers were originally developed using blood methylation data, previous studies have applied markers such as *PhenoAge* and *GrimAge* to non‐blood tissues, including tumour tissue, and observed differences between tumour and normal adjacent tissue after accounting for chronological age and estimated immune cell composition [22]. These findings suggest that these markers may capture methylation variation in tumour tissue that is not solely explained by differences in immune cell composition [22], providing a rationale for assessing their prognostic value in tumour tissue.

Previous studies investigating chronological age markers in tumours and cancer survival have reported varying findings, including no association with mortality in colorectal cancer [18], and a positive association in gliomas [19] and gastric cancer [20] and substantial variation across molecular subtypes of the same cancer [18]. A study of *Horvath age* in breast tumours found, counterintuitively, that younger chronological age was associated with worse prognosis, although the study included only one epigenetic measure, had a small sample size, and did not consider subgroup analysis by molecular subtype [21]. *MRscore* and *PhenoAge* were found to have excellent ability to distinguish between tumour and normal tissue [23] but to our knowledge, studies of their prognostic value are lacking. Regarding markers of cell division, both *MiAge* [9] and *CellDRIFT* [10] measured in breast cancer tumours have been reported to be associated with worse patient survival, albeit based on small sample sizes.

The aims of this study were to (i) compare a series of epigenetic markers for cell division and ageing in breast tumours; and (ii) assess their association with survival, overall and by molecular subtype, to evaluate their potential value as prognostic markers in breast cancer.

## 2 Materials and Methods

### 2.1 Study Sample

We used tumour DNA methylation data and survival information from 425 participants in the Melbourne Collaborative Cohort Study (MCCS), 688 participants in The Cancer Genome Atlas (TCGA), and 879 participants from several datasets available at the Gene Expression Omnibus (GEO) repository, for a total of 1992 participants. The GEO series included GSE37754 (59 patients), GSE69914 (302 patients), GSE72245 (116 patients), GSE72251 (117 patients), GSE72254 (51 patients), GSE75067 (171 patients) and GSE78754 (63 patients).

### 2.2 DNA Methylation Processing and Normalisation

All included studies used the Illumina HumanMethylation450 BeadChip to measure genome‐wide DNA methylation. For five studies with raw data available (IDAT files; MCCS, TCGA, GSE72245, GSE72251 and GSE72254), normalisation and generation of methylation beta values was carried out using the *ENmix* pipeline [24], which includes background correction, dye‐bias correction and probe‐type bias correlation, and has excellent reported performance [25]. For the remaining datasets, we used the normalised data available at GEO. We excluded 416 probes annotated to the Y chromosome as well as 38,941 potential cross‐hybridising probes, as identified by Chen et al. [26] and Benton et al. [27] using the *maxprobes* R package. We further excluded CpG with > 1% missing values across samples in the pooled dataset and the remaining missing values were imputed using the k‐nearest neighbours algorithm.

### 2.3 Epigenetic Markers of Cell Division and Ageing

We considered a total of 10 epigenetic markers of cell division and ageing. These comprised four markers of cell division: *epiTOC2* [7], *stemTOC* [8], *MiAge* [9] and *CellDRIFT* [10]; two chronological age markers: *Horvath age* [11] and *BTEC* [12]; and four biological age markers: *PhenoAge* [13], *GrimAge* [14], *MRscore* [15] and *DunedinPACE* [16]. These were selected for being, to our knowledge, the most widely used and validated markers; it is noted that other valuable markers [28, 29, 30, 31, 32] would partially overlap with those included here. A detailed description of the included markers, including their function, the principles for selecting CpGs, and the training tissue and samples used, as well as a statistical summary of their values calculated in this study, is presented in Table 1.

**TABLE 1:** Summary of the selected DNA methylation‐based markers.

Marker type	Marker	Functions, principles for selecting CpGs and training tissue used	*r* with age	Mean (SD) /median (IQR)
Cell division markers	*EpiTOC2*	*EpiTOC2* estimates the cumulative number of stem cell divisions per stem cell in a tissue. The model used the same set of CpG sites from *epiTOC*, a previously developed model that estimates the rate of stem cell divisions in a tissue. CpG sites were selected to be unmethylated across foetal tissues and to map to Polycomb group target promoters. In addition, methylation levels at the selected CpG sites were required to increase with chronological age. A total of 163 CpG sites were used to calculate *epiTOC2*. The model was developed using whole blood samples from healthy participants across a wide age range [7, 33].	0.23	8222 (6272 to 10,898)
	*StemTOC*	*StemTOC* estimates the cumulative number of stem cell divisions per stem cell in a tissue. CpG sites were selected to be constitutively unmethylated across foetal and neonatal tissue types, reducing potential confounding by cell‐type heterogeneity. To reduce confounding by chronological age, selected CpG sites were required to show increasing methylation with increasing population doublings across multiple normal in vitro cell lines, but not when these cell lines were growth arrested or cultured under reduced growth‐promoting conditions. To reduce the possibility of cell‐culture effects, selected CpG sites were also required to show age‐associated methylation gains in in vivo whole‐blood datasets. A total of 371 CpG sites were selected to calculate *stemTOC*. The model was developed using foetal and neonatal tissue types, normal cell lines and in vivo whole‐blood methylation datasets [8].	0.20	0.56 (0.46 to 0.65)
	*MiAge*	*MiAge* estimates the total number of lifetime cell divisions in a tissue, up to a proportionality constant. CpG sites were selected to be informative of mitotic activity and not tissue or tumour specific. CpG sites were required to show increasing methylation with cell division. A total of 268 CpG sites were selected to calculate *MiAge*. The model was developed using tumour and adjacent normal tissues from eight TCGA cancer types [9].	0.21	1238 (867 to 1629)
	*CellDRIFT*	*CellDRIFT* quantifies a DNAm‐based replication signature in tissues. CpG sites were selected based on their associations with cumulative population doublings in immortalised human foetal astrocytes and their consistency across in vitro and in vivo tissues. A total of 2322 CpG sites were selected to calculate *CellDRIFT*. The model was developed using extensively passaged hTERT immortalised human foetal astrocytes [10].	0.21	44 (41 to 47)
Chronological age markers	*Horvath age*	*Horvath age* is a pan‐tissue predictor of chronological age. CpG sites were selected based on their associations with chronological age using a penalised regression approach. A total of 353 CpG sites were selected to calculate *Horvath age*. The model was developed using non‐cancer samples from 51 different tissues and cell types [11].	0.43	64 (21)
	*BTEC*	*BTEC* predicts chronological age in breast tissue. CpG sites were selected based on their associations with chronological age. A total of 799 CpG sites were selected to calculate *BTEC*. The model was developed using breast tissues from healthy individuals with ages ranging from 13 to 90 years [12].	0.34	42 (25)
Biological age markers	*PhenoAge*	*PhenoAge* predicts phenotypic age, a composite measure designed to capture differences in mortality risk among individuals of the same chronological age. Phenotypic age was first estimated in a nationally representative sample using nine clinical biomarkers, chronological age and sex, selected to optimise prediction of mortality risk. Elastic net regression was then used to select a total of 513 CpG sites to predict phenotypic age. The model was developed using whole blood samples [13].	0.34	76 (59 to 95)
	*GrimAge*	*GrimAge* is a predictor of all‐cause mortality. It was constructed using an elastic net Cox regression model based on chronological age, sex, a DNAm‐based estimator of smoking pack‐years, and seven DNAm‐based surrogate markers of plasma proteins. A total of 1030 CpG sites were used to calculate *GrimAge*. The model was developed using whole blood samples [14].	0.84	86 (11)
	*MRscore*	*MRscore* predicts all‐cause mortality. In an epigenome‐wide association study, 58 CpG sites were identified based on their associations with all‐cause mortality. LASSO regression was then applied to select 10 CpG sites to construct a mortality risk score, either as a weighted continuous score or as a categorical score based on the number of CpG with aberrant methylation. The model was developed using whole blood samples [15].	0.19	−0.74 (−1.32 to −0.32)
	*DunedinPACE*	*DunedinPACE* measures the ongoing rate at ageing. CpG sites were selected based on their ability to predict the Pace of Ageing, which was derived from longitudinal changes in 19 indicators of organ‐system integrity across cardiovascular, metabolic, renal, hepatic, pulmonary, periodontal and immune systems. A total of 173 CpG sites were selected to calculate *DunedinPACE*. The model was developed using whole blood samples from the Dunedin Study cohort [16].	0.10	1.72 (0.29)

Abbreviations: DNAm, DNA methylation; IQR, interquartile range; *r*, correlation coefficient; SD, standard deviation.

All exposures were considered relative to age, so for each epigenetic marker, the corresponding measure was calculated as the residuals from a linear regression of the epigenetic marker on chronological age [31]. Each measure was further scaled to have a mean of zero and a standard deviation of one to allow better comparison of results across markers on very different scales (Table 1).

### 2.4 Survival Outcome

Five‐year overall survival was chosen as the outcome because it was the only well‐defined endpoint across all datasets. Time at risk of death was calculated from the date of diagnosis to the date of death, or the end of follow‐up; participants were censored at 5 years postdiagnosis.

### 2.5 Confounders

When assessing the associations between epigenetic markers and 5‐year overall survival, several potential confounders were identified a priori, including age at diagnosis, ER status, molecular subtype (Luminal A, Luminal B, HER2‐positive and triple‐negative), tumour stage, tumour grade and tumour purity (estimated based on the methylation data using the *InfiniumPurify* R package [17]). Although age‐adjusted epigenetic markers are, by definition, independent of chronological age, age at diagnosis was still considered as a confounder for being both a biological and a social variable [34]. Previous studies have reported that ER status [35], molecular subtype [12], tumour stage [18] and tumour purity [8] are likely to be reflected by epigenetic processes. Tumour grade is essentially an indicator of tumour growth and was also considered as a potential confounder; compared with low‐grade tumours, high‐grade tumours exhibit greater methylation alterations [36].

### 2.6 Statistical Analysis

Pearson correlation coefficients among raw and age‐adjusted epigenetic markers, chronological age and tumour purity were calculated; two correlation matrices were presented, one for the raw epigenetic markers and one for the age‐adjusted epigenetic markers.

The associations between epigenetic markers and clinical variables, including ER status, molecular subtype (Luminal A, Luminal B, HER2‐positive and triple‐negative), tumour stage (I, II, and III/IV), and tumour grade (1, 2 and 3), were evaluated using linear regression models. In this analysis, epigenetic markers were treated as outcomes, and clinical variables as exposures. ER status and molecular subtype were treated as categorical exposures, whereas tumour stage and tumour grade were treated as pseudo‐continuous variables. Both univariate and multivariable models (all variables mentioned above) were fitted. Because not all participants had complete information on molecular subtype, tumour stage and tumour grade, multivariable analyses and corresponding univariate analyses were restricted to participants with complete data on clinical variables.

Cox regression models were used to estimate hazard ratios (HRs) and their 95% confidence intervals for the associations of age‐adjusted epigenetic markers with 5‐year risk of overall death. Two models were fitted: Model 1 adjusted for age and stratified by study, allowing the baseline hazard rate to vary by study while assuming a common HR for associations of epigenetic markers with survival across studies [37]. Model 2 added to Model 1 ER status, and the interaction between age and ER status, as the association between age and breast cancer survival may vary by ER status, in part due to typically aggressive, ER‐negative tumours diagnosed at younger ages [38]. All 1992 participants had complete data on age and ER status. Subgroup analyses using Model 1 were carried out separately for ER‐positive and ER‐negative tumours, as well as for each molecular subtype. Effect modification by ER status and molecular subtype was assessed using likelihood ratio tests of models with and without interaction terms between epigenetic markers and ER status, and molecular subtype, respectively.

#### 2.6.1 Sensitivity Analysis

A sensitivity analysis was conducted to assess associations after adjusting for additional clinical characteristics, including molecular subtype, tumour stage, tumour grade and tumour purity. Most MCCS participants had complete information for these variables, whereas TCGA participants lacked tumour grade data and participants from the GEO datasets lacked tumour stage data. We carried out a complete‐case analysis for these variables as available in each respective dataset. A Cox regression model was fitted for the MCCS participants with complete clinical data, adjusting for age, ER status and its interaction with age, molecular subtype, tumour stage, tumour grade and tumour purity. For 439 TCGA participants with complete data on molecular subtype and tumour stage, the model included all variables except tumour grade. Similarly, for 505 GEO participants with complete data on molecular subtype and tumour grade, the model included all variables except tumour stage. For each epigenetic marker, the results from MCCS, TCGA and GEO datasets were then pooled using fixed‐effects meta‐analysis with inverse‐variance weights (Figure S1). The pooled results were compared against Model 2 fitted again for the same set of 1347 participants included in the sensitivity analysis to ensure that any differences between the two sets of results reflected the inclusion of additional clinical variables rather than sampling differences.

All statistical analyses were performed using R (version 4.5.0).

## 3 Results

### 3.1 Sample Characteristics

The demographic and clinicopathological characteristics of the 1992 breast cancer patients are shown in Table 2. MCCS participants were older (mean age: 63.9 years, SD: 8.4) than those from TCGA (57.8 years, SD: 12.9) and GEO datasets (56.0 years, SD: 13.4). ER status was positive for 69.1% of women overall, the percentage being highest in the MCCS (80.2%) and lowest in GEO (57.7%), reflecting enrichment of triple‐negative cases in some of these datasets. For molecular subtype, among participants with available data, Luminal A was the most common across studies (38.7%), with HER2‐positive being the least common (5.5%). Most tumours in the MCCS were stage I (57.9%), whereas most tumours in TCGA were stage II (55.1%). Tumour purity estimates were similar across studies, with an overall mean of 60% (SD: 14%).

**TABLE 2:** Sample demographic and clinicopathological characteristics (*N* = 1992).

	MCCS (*N* = 425)	TCGA (*N* = 688)	GEO (*N* = 879)	Pooled (*N* = 1992)
Age (years), mean (SD)	63.9 (8.4)	57.8 (12.9)	56.0 (13.4)	58.3 (12.7)
ER status, *n* (%)				
Negative	84 (19.8%)	159 (23.1%)	372 (42.3%)	615 (30.9%)
Positive	341 (80.2%)	529 (76.9%)	507 (57.7%)	1377 (69.1%)
Molecular subtype, *n* (%)				
Luminal A	242 (56.9%)	288 (41.9%)	241 (27.4%)	771 (38.7%)
Luminal B	87 (20.5%)	62 (9.0%)	92 (10.5%)	241 (12.1%)
HER2‐positive	31 (7.3%)	14 (2.0%)	64 (7.3%)	109 (5.5%)
Triple‐negative	65 (15.3%)	78 (11.3%)	172 (19.6%)	315 (15.8%)
Missing	0 (0%)	246 (35.8%)	310 (35.3%)	556 (27.9%)
Tumour stage, *n* (%)				
I	246 (57.9%)	116 (16.9%)	0 (0%)	362 (18.2%)
II	135 (31.8%)	379 (55.1%)	0 (0%)	514 (25.8%)
III/IV	44 (10.4%)	187 (27.2%)	0 (0%)	231 (11.6%)
Missing	0 (0%)	6 (0.9%)	879 (100%)	885 (44.4%)
Tumour grade, *n* (%)				
1	89 (20.9%)	0 (0%)	121 (13.8%)	210 (10.5%)
2	174 (40.9%)	0 (0%)	236 (26.8%)	410 (20.6%)
3	140 (32.9%)	0 (0%)	433 (49.3%)	573 (28.8%)
Missing	22 (5.2%)	688 (100%)	89 (10.1%)	799 (40.1%)
Tumour purity (%), mean (SD)	62 (12)	66 (14)	55 (12)	60 (14)
Median survival, (years)	15.5	2.4	5.8	5.6
Deaths within 5 years, *n* (%)	47 (11)	63 (9)	162 (18)	272 (14)

Abbreviations: ER, estrogen receptor; SD, standard deviation.

### 3.2 Correlations Between Epigenetic Markers

**FIGURE 1:**
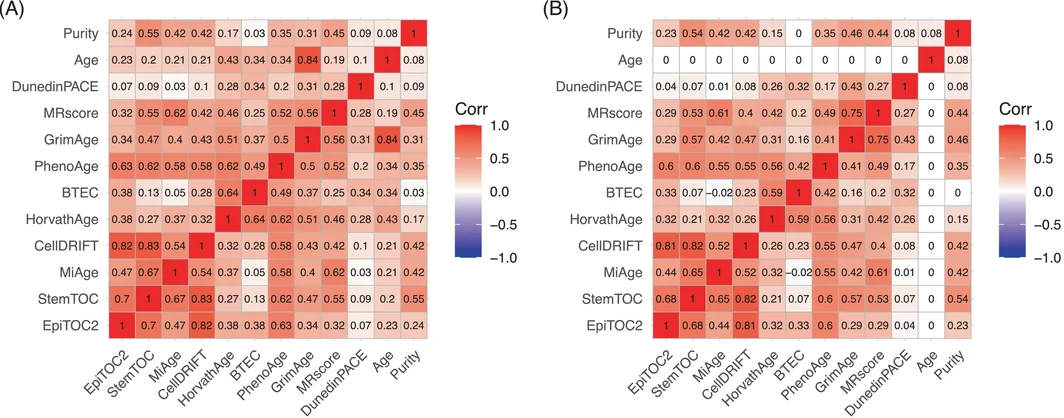
Correlation matrix of epigenetic markers, chronological age and tumour purity (*N* = 1992). (A) Correlations calculated using raw values of epigenetic markers. (B) Correlations calculated using age‐adjusted values of epigenetic markers.

Overall, the three types of epigenetic markers (cell division, chronological age and biological age) were moderately correlated with each other, except for the biological age marker *DunedinPACE*, which showed low correlations with all other markers (Figure 1A). Moderate to strong correlations were observed among cell division markers, that is, *epiTOC2*, *stemTOC*, *MiAge* and *CellDRIFT*, with correlation coefficients ranging from *r* = 0.47 to 0.83. A fairly strong correlation was observed between the chronological age markers, *Horvath age* and *BTEC* (*r* = 0.64). Among the biological age markers, *PhenoAge*, *GrimAge* and *MRscore* showed moderate correlations, ranging from 0.50 to 0.56. Tumour purity estimates showed weak to moderate correlations with most epigenetic markers and was highest for the cell division marker *stemTOC* (*r* = 0.55). The pattern of correlations between epigenetic markers was similar when considering the age‐adjusted measures (Figure 1B), although the correlation between *GrimAge* and *MRscore* strengthened (*r* = 0.75, up from *r* = 0.56).

### 3.3 Cross‐Sectional Associations Between Epigenetic Markers and Clinical Variables

**FIGURE 2:**
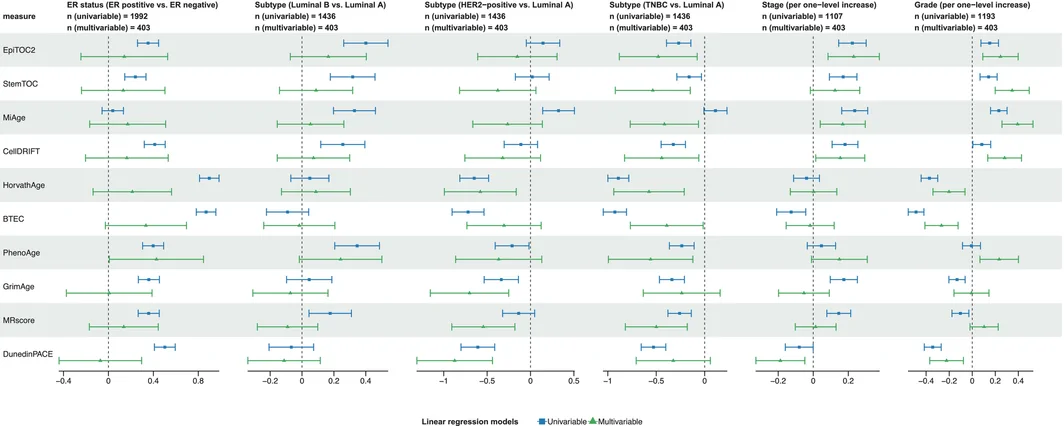
Associations between epigenetic markers and clinical variables. Linear regression analyses were conducted to evaluate the associations between epigenetic markers, treated as outcomes, and clinical variables, which were treated as exposures. ER status and subtype were treated as categorical variables, whereas stage and grade were treated as ordinal variables. Of the total 1992 participants, all were included in the univariable analysis between ER status and epigenetic markers; 1436 participants were included in the univariable analysis between molecular subtype and epigenetic markers; 1107 participants were included in the univariable analysis between tumour stage and epigenetic markers; and 1193 participants were included in the univariable analysis between tumour grade and epigenetic markers. A total of 403 participants from the MCCS were included in the multivariable analyses, each adjusted for other clinical variables. Each epigenetic marker was scaled to have a mean of zero and a standard deviation of one. Abbreviations: ER, estrogen receptor; TNBC, triple‐negative breast cancer.

Markers of cell division were generally greater in ER‐positive tumours compared with ER‐negative tumours, except for *MiAge* (Figure 2), with fairly strong attenuation after adjustment for other clinical variables. Luminal B tumours exhibited higher values of cell division markers, and triple‐negative tumours generally lower values. Both tumour stage and tumour grade were positively associated with the four markers of cell division in both univariable and multivariable analyses.

ER‐negative tumours showed higher values of the chronological age markers *Horvath age* and *BTEC*. HER2‐positive and triple‐negative tumours generally exhibited lower values than Luminal A and Luminal B tumours. These markers showed no associations with tumour stage and a negative association with tumour grade.

Biological age markers generally showed lower values in Luminal B, HER2‐positive and triple‐negative tumours compared with Luminal A tumours. *DunedinPACE* showed negative associations with both tumour stage and tumour grade, whereas other biological age markers showed positive or null associations.

### 3.4 Prospective Associations Between Epigenetic Markers and 5‐Year Overall Survival

**FIGURE 3:**
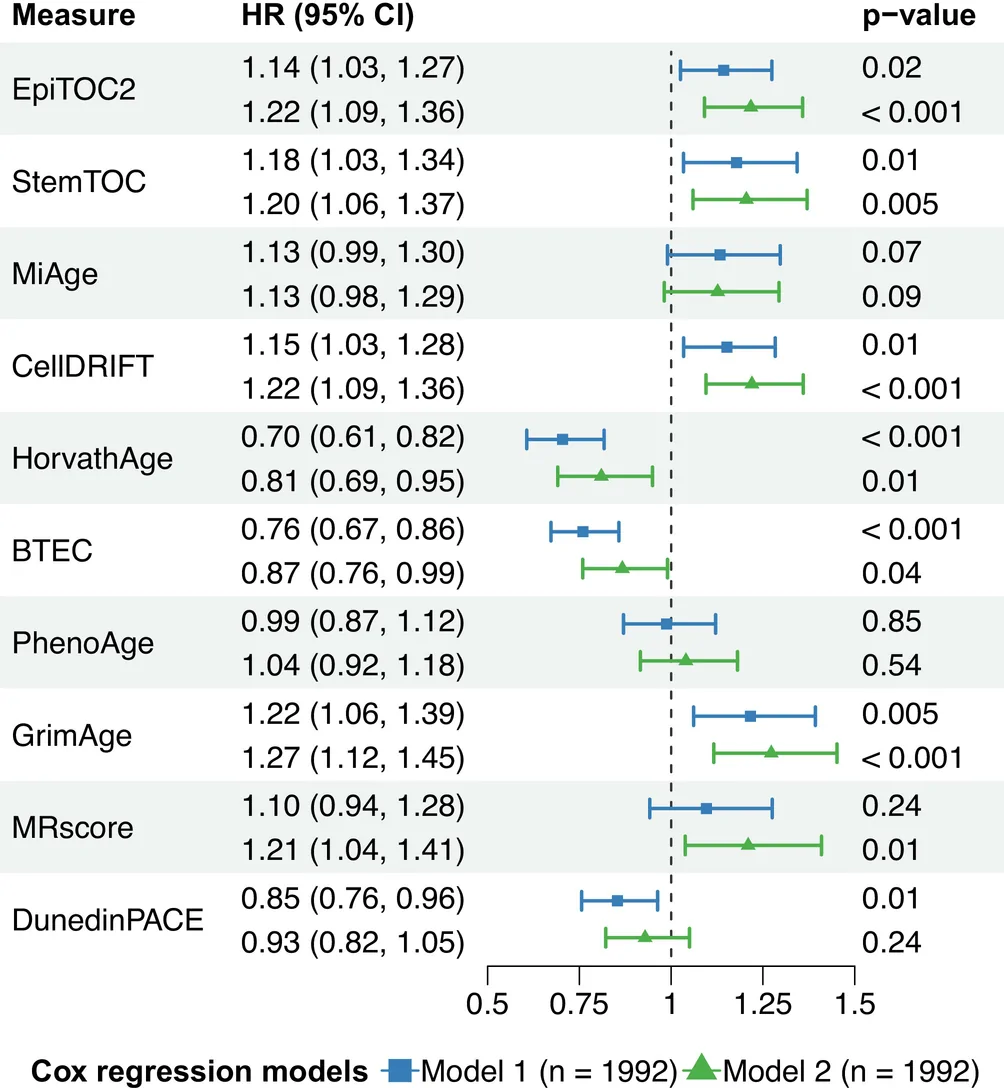
Associations between epigenetic markers and 5‐year overall survival (*N* = 1992; *N* deaths = 272). Model 1 was adjusted for age and stratified by study. Model 2 was adjusted for age, ER status, and the interaction between age and ER status and was stratified by study. Each epigenetic marker was scaled to have a mean of zero and a standard deviation of one. Abbreviations: CI, confidence interval; ER, estrogen receptor; HR, hazard ratio.

In Model 1, adjusting for age, markers of cell division, chronological age and biological age showed distinct patterns in relation to risk of death (Figure 3). *EpiTOC2* (HR = 1.14, 95% CI: 1.03–1.27, *p* = 0.02), *stemTOC* (HR = 1.18, 95% CI: 1.03–1.34, *p* = 0.01), *CellDRIFT* (HR = 1.15, 95% CI: 1.03–1.28, *p* = 0.01) and *GrimAge* (HR = 1.22, 95% CI: 1.06–1.39, *p* = 0.005) were positively associated with risk of death. *Horvath age* was associated with longer survival (HR = 0.70, 95% CI: 0.61–0.82, *p* < 0.001), as was the other chronological age marker *BTEC* (HR = 0.76, 95% CI: 0.67–0.86, *p* < 0.001), and the biological age marker *DunedinPACE* (HR = 0.85, 95% CI: 0.76–0.96, *p* = 0.01). The associations were generally similar in Model 2, which additionally adjusted for ER status and the interaction between age and ER status, with *MRscore* showing a stronger positive association with risk of death (HR = 1.21, 95% CI: 1.04–1.41, *p* = 0.01) than in Model 1 (HR = 1.10, 95% CI: 0.94–1.28, *p* = 0.24). The association of *DunedinPACE* was attenuated towards the null (HR = 0.93, 95% CI: 0.82–1.05, *p* = 0.24).

In the subgroup analysis by ER status, the associations tended to be stronger in ER‐positive tumours than in ER‐negative tumours (Table S1). In ER‐positive tumours, *epiTOC2* was strongly associated with risk of death (HR = 1.38, 95% CI: 1.19–1.61, *p* < 0.001). There was evidence of effect modification by ER status (P‐interaction = 0.02). *PhenoAge*, *GrimAge* and *MRscore* also showed substantially greater HR estimates in ER‐positive tumours though without evidence of effect modification (*p* > 0.05). In analyses by molecular subtype, all markers of cell division showed positive associations in HER2‐positive tumours, with *epiTOC2* (HR = 1.93, 95% CI: 1.11–3.35, *p* = 0.02) and *stemTOC* (HR = 2.04, 95% CI: 1.11–3.75, *p* = 0.02) showing the strongest associations (Table S2). In contrast, chronological age markers showed the strongest, negative associations in Luminal B tumours, for both *Horvath age* (HR = 0.67, 95% CI: 0.43–1.04, *p* = 0.08) and *BTEC* (HR = 0.58, 95% CI: 0.39–0.86, *p* = 0.01), with evidence of effect modification for *BTEC* (*p* = 0.02). Biological age markers showed varying associations with survival across molecular subtypes.

#### 3.4.1 Sensitivity Analysis

**FIGURE 4:**
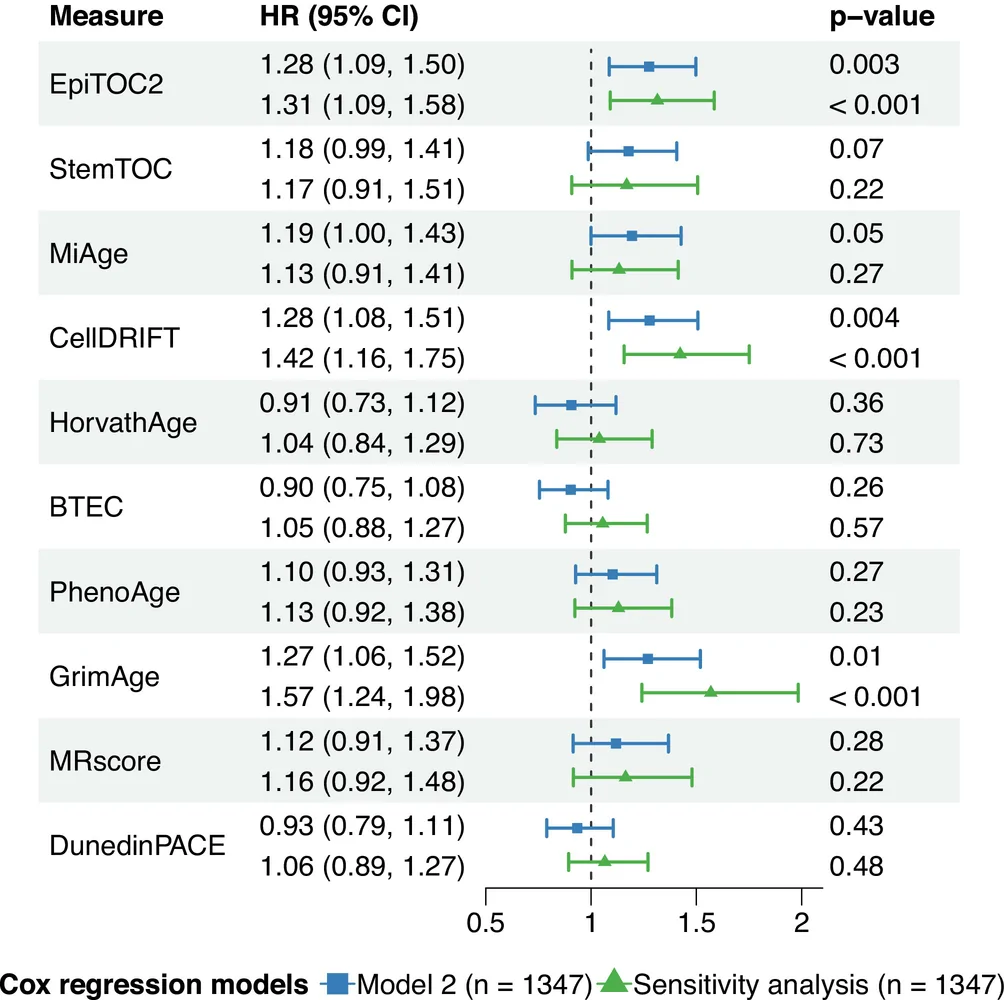
Sensitivity analysis for the association between epigenetic markers and 5‐year overall survival (*N* = 1347; *N* deaths = 143). For each epigenetic measure, the results from MCCS, TCGA and GEO datasets were pooled using a fixed‐effects meta‐analysis with inverse‐variance weights, which formed the results for the sensitivity analysis adjusting for age, ER status, the interaction between them, molecular subtype, tumour stage, tumour grade and tumour purity. The results from Model 2 fitted on the same set of participants were then compared with these. Each epigenetic marker was scaled to have a mean of zero and a standard deviation of one. Abbreviations: CI, confidence interval; ER, estrogen receptor; HR, hazard ratio.

When Model 2 was restricted to the 1347 participants included in the sensitivity analysis, the overall direction of the associations remained the same as when fitted to all participants (Figures 3 and 4). Some attenuation towards the null was observed for most associations in Model 2 fitted to the 1347 participants (Figure 4), except for *epiTOC2*, *CellDRIFT* and *GrimAge*. After adjustment for more clinical characteristics, the associations were attenuated for several markers but were greater than in Model 2 for *epiTOC2* (HR = 1.31, 95% CI: 1.09–1.58, *p* < 0.001), *CellDRIFT* (HR = 1.42, 95% CI: 1.16–1.75, *p* < 0.001) and *GrimAge* (HR = 1.57, 95% CI: 1.24–1.98, *p* < 0.001).

## 4 Discussion

This study examined three types of DNA methylation‐based epigenetic markers measured in breast cancer tissues in relation to patient clinical characteristics and 5‐year overall survival. Markers of cell division, chronological age and biological age were applied in a large sample of breast tumours with genome‐wide DNA methylation measurements.

Moderate to strong correlations were observed within each class of markers, likely reflecting their shared biological functions as well as the influence of chronological age [9]. Different patterns in the associations between epigenetic markers and clinical variables were observed. HER2‐positive tumours showed lower chronological age markers than Luminal A tumours, whereas cell division markers did not differ between these two subtypes. Both classes of markers were decreased in triple‐negative tumours relative to Luminal A tumours. Tumour grade and tumour stage showed similar patterns, with higher values linked to increased cell division markers but decreased chronological age markers. The associations with mortality varied in both strength and direction across markers, with cell division markers being linked to shorter survival, and chronological age markers to longer survival. Associations for biological age markers were less consistent across markers, although both *GrimAge* and *MRscore* showed a positive association with mortality. Some variation by ER status and molecular subtype was also suggested. Associations tended to be stronger in ER‐positive tumours than in ER‐negative tumours, although formal statistical evidence of effect modification was limited. Cell division markers showed the strongest associations in HER2‐positive tumours, whereas chronological age markers showed the strongest associations in Luminal B tumours. Within the same sample with complete data on the main clinical characteristics, the associations were generally robust to more comprehensive adjustment and were in fact greater for *epiTOC2*, *CellDRIFT* and *GrimAge*. Tumour stage, tumour grade and molecular subtype reflect macroscopic and histopathological characteristics of breast cancer [39, 40], whereas epigenetic markers capture the underlying molecular alterations within tumour cells [7, 8, 9, 10, 11, 12, 13, 14, 15, 16]. These clinical variables and epigenetic markers are likely to represent, to some extent, different levels of the same process. Therefore, the stronger associations observed for *epiTOC2*, *CellDRIFT* and *GrimAge* suggest that these epigenetic markers may provide additional prognostic information. Although some epigenetic markers were associated with overall survival in this sample, associations were generally weak and may be unlikely to translate into meaningful survival prediction gains that could impact clinical practice.

The positive associations between the markers considered in this study and cancer mortality are consistent with some findings reported in the context of other cancer types. Markers of cell division have been associated with worse prognosis in prostate cancer [28], oral squamous cell carcinoma [41] and chronic lymphocytic leukaemia [42], whereas associations with better survival have been reported in gliomas [43]. These varying results suggest that associations between cell division markers and survival may differ by cancer type, reflecting the influence of underlying biological processes specific to different tumour tissues. Aggressive gliomas are characterised by high transcriptional heterogeneity and plasticity, allowing tumour cells to shift away from highly proliferative states due to low transition barriers between cell states [44]. This phenomenon may explain the negative associations of cell division markers with mortality found for gliomas. Collectively, these observations indicate that the associations between cell division markers and survival may depend on the specific cancer context.

Chronological age markers have also shown mixed associations with survival across tumour types [18, 19, 20]. Higher *Horvath age* has been linked to shorter survival when measured in colorectal tumours [18] but to longer survival in gliomas and gastric cancer tissues [19, 20]. One study examining breast cancer tissues reported a negative association between *Horvath age* and mortality [21], consistent with our findings. This negative association with mortality observed for chronological age markers in our study and other studies could be explained by tumour dedifferentiation, a phenomenon characteristic of aggressive tumours. During dedifferentiation, tumour cells become less specialised and revert to an undifferentiated and stem‐cell‐like state [44]. Therefore, a decrease in chronological age markers in tumour tissues may indicate the existence of tumour dedifferentiation, an event linked to aggressive tumours with worse prognosis, as opposed to more differentiated and less stem‐cell‐like tumours. In our study, decreases in chronological age markers were observed for HER2‐positive, triple‐negative and high‐grade tumours compared to other less aggressive tumours, consistent with a greater degree of tumour dedifferentiation in these subtypes.

A major strength of this study is the application of three types of well‐validated epigenetic markers to breast tumour tissues in a large number of patients with survival information; to our knowledge, this pooled analysis had the largest sample size to date for research of this kind. This design enabled direct comparison of the prognostic potential of these markers and provided an opportunity to examine how different classes of markers captured distinct biological processes within breast tumours. The contrasting patterns observed between cell division markers and chronological age markers allowed us to link specific tumour characteristics to the underlying biological processes captured by these epigenetic measures.

Several limitations should be noted. Because this was a pooled analysis, not all studies collected the same set of clinical variables, which limited our ability to perform fully comprehensive adjustment of the survival analyses. Nonetheless, the sensitivity analysis confirmed that our findings were overall robust to the inclusion of additional clinical variables and sampling variability. In addition, combining data across studies likely increased statistical and biological variability, for example in terms of the study sample characteristics or technical aspects related to the measurement of genome‐wide DNA methylation in tumours. Another limitation is that for most of the included cohorts, breast cancer‐specific outcomes were not available. As a result, our analysis was restricted to overall survival, which may include deaths unrelated to breast cancer and therefore result in underestimated associations. This calls for subsequent studies evaluating associations with breast cancer‐specific outcomes to better evaluate the prognostic potential of these epigenetic markers. A further limitation is that the interpretation of blood‐based biological age markers in tumour tissue is challenging. Originally developed in blood, *GrimAge* and *PhenoAge* strongly correspond to ageing‐associated risk of disease and mortality [13, 14]. When applied to tumour tissue, these markers may also capture immune cell infiltration and surveillance activities that suppress tumour growth, in addition to tissue ageing. Future studies are therefore required to clarify the biological processes captured by these clocks in tumours, likely involving a combination of age‐related and immune‐related changes, as well as overall aberrant epigenetic alterations inherent to carcinogenesis.

## 5 Conclusion

This study revealed associations between several epigenetic markers in tumours and mortality following breast cancer, yet variations across ER status and molecular subtypes suggest that their prognostic utility may depend on disease subtype. Several epigenetic markers of both cell division and ageing appeared to capture molecular processes not reflected by traditional clinical variables such as tumour stage and tumour grade. Future research should consider subtype‐specific studies and incorporate breast cancer‐specific outcomes to more fully determine the prognostic value of these epigenetic markers beyond conventional clinical variables.

## Author Contributions

**Luca Li:** conceptualization, methodology, formal analysis, writing – original draft. **Elaheh Zarean:** conceptualization, data curation, writing – review and editing. **Danmeng Lily Li:** conceptualization, supervision, writing – review and editing. **Yiyue Zhu:** conceptualization, data curation, writing – review and editing. **Shuai Li:** conceptualization, writing – review and editing. **Enes Makalic:** conceptualization, writing – review and editing. **Catriona McLean:** conceptualization, data curation, writing – review and editing. **Graham G. Giles:** conceptualization, resources, funding acquisition, writing – review and editing. **Roger L. Milne:** conceptualization, methodology, resources, writing – review and editing. **Melissa C. Southey:** conceptualization, data curation, funding acquisition, resources, writing – review and editing. **Pierre‐Antoine Dugué:** conceptualization, methodology, data curation, supervision, formal analysis, writing – original draft.

## Funding

Melbourne Collaborative Cohort Study (MCCS) cohort recruitment was funded by VicHealth and Cancer Council Victoria. The MCCS was further supported by Australian National Health and Medical Research Council (NHMRC) grants 209057, 396414 and 1074383 and by infrastructure provided by Cancer Council Victoria. Cases and their vital status were ascertained through the Victorian Cancer Registry. This research also received funding from NHMRC grant 1011618. M.C.S. is supported by an NHMRC L3 Investigator Fellowship (GNT2017325), P.‐A.D. by a Victoria Cancer Agency Mid‐Career Fellowship (MCRF22025) and S.L. by an NHMRC EL1 Investigator Fellowship (GNT2017373). E.Z. and D.L.L. are supported by a Monash University PhD scholarship.

## Ethics Statement

The study was conducted according to the guidelines of the Declaration of Helsinki and approved by the Human Research Ethics Committee of Cancer Council Victoria (protocol code IEC #9001, 31 December 1990).

## Consent

All participants provided informed consent before participating in the study.

## Conflicts of Interest

The authors declare no conflicts of interest.

## Supporting information


**Figure S1:** Forest plot for the sensitivity analysis using complete‐case data.
**Table S1:** Subgroup analysis by ER status for the associations between epigenetic markers and 5‐year overall survival (*n* = 1992; *n* deaths = 272).
**Table S2:** Subgroup analysis by molecular subtype for the associations between epigenetic markers and 5‐year overall survival (*n* = 1436; *n* deaths = 172).

## Data Availability

The TCGA‐BRCA data used in this study are publicly available at https://www.cancer.gov/tcga, generated by the TCGA Research Network. The GEO data sets used in this study are publicly available at the NCBI Gene Expression Omnibus (https://www.ncbi.nlm.nih.gov/geo/) under accession numbers GSE37754, GSE69914, GSE72245, GSE72251, GSE72254, GSE75067 and GSE78754. The MCCS individual participant data is under ethical protection, and can be available by requesting the PEDIGREE resource via pedigree@cancervic.org.au. Further data that support the findings of this study are available from the corresponding author upon request.
