# Supplementary material for: Epigenetic Markers of Cell Division and Ageing in Relation to Breast Cancer Survival
Source: Int J Cancer. 2026 Jun 27;159(10):2452–62. doi: 10.1002/ijc.70618 (PMC13595474; doi:10.1002/ijc.70618)
Supplement: Supplementary file 1 [file IJC-159-2452-s001.pdf]

SUPPLEMENTARY MATERIAL

Epigenetic markers of cell division and ageing in relation to breast cancer survival

Luca Li, Elaheh Zarean, Danmeng Lily Li, Yiyue Zhu, Shuai Li, Enes Makalic, Catriona McLean, Graham G. Giles, Roger L. Milne, Melissa C. Southey, Pierre-Antoine Dugué

Table of Contents

Figure S1. Forest plot for the sensitivity analysis using complete-case data. 2

Table S1. Subgroup analysis by ER status for the associations between epigenetic markers and five-year overall survival (n = 1,992; n deaths = 272). 3

Table S2. Subgroup analysis by molecular subtype for the associations between epigenetic markers and five-year overall survival (n = 1436; n deaths = 172). 4

Figure S1. Forest plot for the sensitivity analysis using complete-case data.

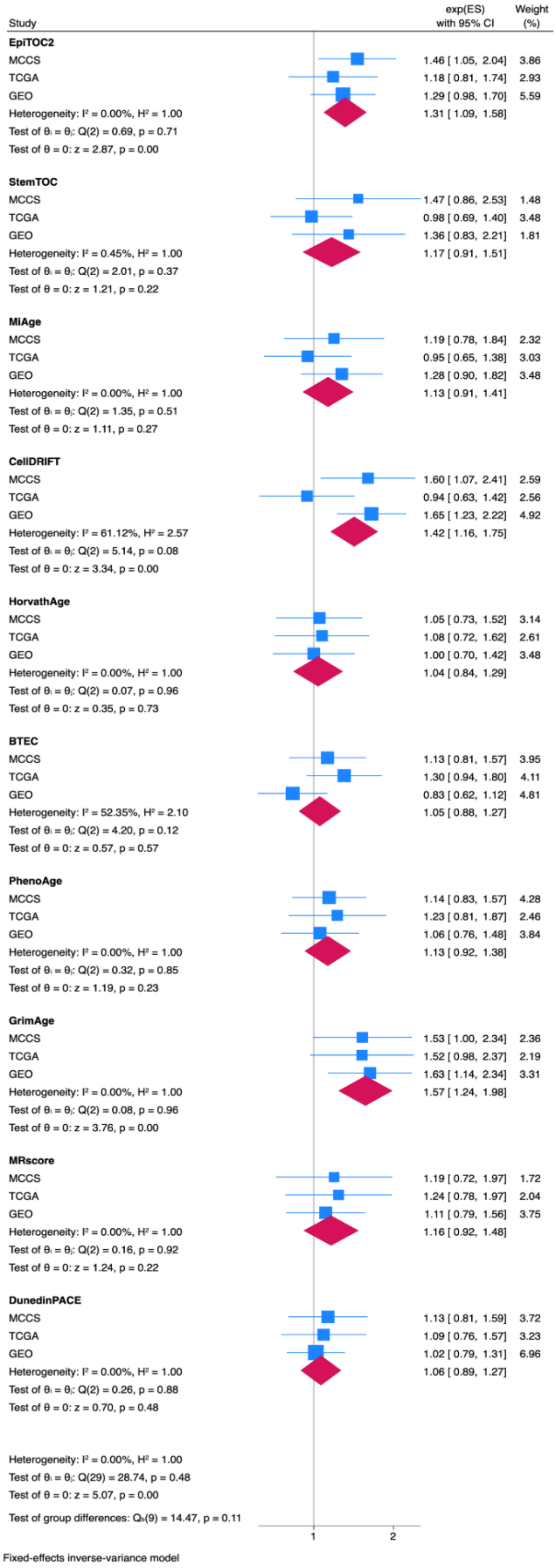

Table S1. Subgroup analysis by ER status for the associations between epigenetic markers and five-year overall survival (n = 1,992; n deaths = 272).

	**ER-negative (n = 615; n deaths = 149)**	**ER-positive (n = 1377; n deaths = 123)**	
**Epigenetic markers**	**HR (95% CI)**	**HR (95% CI)**	***p*-value**[a]
EpiTOC2	1.06 (0.90 to 1.26)	1.38 (1.19 to 1.61)	0.02
StemTOC	1.12 (0.94 to 1.33)	1.35 (1.11 to 1.64)	0.11
MiAge	1.01 (0.82 to 1.26)	1.22 (1.01 to 1.46)	0.19
CellDRIFT	1.17 (1.01 to 1.36)	1.28 (1.09 to 1.51)	0.33
Horvath age	0.78 (0.62 to 0.98)	0.84 (0.67 to 1.05)	0.73
BTEC	0.94 (0.78 to 1.14)	0.81 (0.67 to 0.97)	0.11
PhenoAge	0.93 (0.76 to 1.13)	1.13 (0.96 to 1.34)	0.12
GrimAge	1.24 (1.06 to 1.45)	1.38 (1.08 to 1.76)	0.40
MRscore	1.15 (0.96 to 1.39)	1.32 (1.02 to 1.71)	0.44
DunedinPACE	0.97 (0.83 to 1.14)	0.87 (0.72 to 1.06)	0.30

[a] From the likelihood ratio test assessing effect modification by ER status on the association between each epigenetic marker and mortality hazard.
Each epigenetic marker was scaled to have a mean of zero and a standard deviation of one. The association between each marker and survival was adjusted for age and stratified by study.
Abbreviation: ER, estrogen receptor; HR, hazard ratio; CI, confidence interval.

Table S2. Subgroup analysis by molecular subtype for the associations between epigenetic markers and five-year overall survival (n = 1436; n deaths = 172).

	**Luminal A (n = 771; n deaths = 49)**	**Luminal B (n = 241; n deaths = 26)**	**HER2-positive (n = 109; n deaths = 16)**	**TNBC (n = 315; n deaths = 81)**	
**Epigenetic markers**	**HR (95% CI)**	**HR (95% CI)**	**HR (95% CI)**	**HR (95% CI)**	***p*-value[a]**
EpiTOC2	1.31 (1.03 to 1.67)	1.34 (0.93 to 1.95)	1.93 (1.11 to 3.35)	1.12 (0.90 to 1.39)	0.26
StemTOC	1.16 (0.87 to 1.57)	1.27 (0.78 to 2.08)	2.04 (1.11 to 3.75)	1.16 (0.92 to 1.46)	0.51
MiAge	1.27 (0.98 to 1.65)	1.19 (0.77 to 1.84)	1.29 (0.86 to 1.95)	1.07 (0.81 to 1.41)	0.92
CellDRIFT	1.27 (0.99 to 1.63)	1.24 (0.83 to 1.86)	1.72 (0.95 to 3.14)	1.22 (1.01 to 1.48)	0.81
Horvath age	1.12 (0.81 to 1.56)	0.67 (0.43 to 1.04)	0.81 (0.38 to 1.75)	0.92 (0.70 to 1.21)	0.35
BTEC	1.04 (0.77 to 1.40)	0.58 (0.39 to 0.86)	0.85 (0.42 to 1.71)	1.09 (0.84 to 1.41)	0.02
PhenoAge	1.20 (0.93 to 1.54)	0.88 (0.59 to 1.30)	1.31 (0.64 to 2.66)	1.10 (0.87 to 1.39)	0.40
GrimAge	1.27 (0.87 to 1.87)	1.35 (0.84 to 2.17)	1.52 (0.89 to 2.58)	1.20 (1.00 to 1.45)	0.69
MRscore	1.42 (0.96 to 2.10)	1.18 (0.68 to 2.04)	1.36 (0.68 to 2.73)	1.09 (0.87 to 1.36)	0.76
DunedinPACE	1.07 (0.78 to 1.47)	0.58 (0.38 to 0.87)	0.76 (0.47 to 1.23)	1.04 (0.84 to 1.29)	0.07

[a] From the likelihood ratio test assessing effect modification by molecular subtype on the association between each epigenetic marker and mortality hazard.
Each epigenetic marker was scaled to have a mean of zero and a standard deviation of one. The association between each marker and survival was adjusted for age and stratified by study.
Abbreviation: HR, hazard ratio; CI, confidence interval.
